# What’s the Mass? The Gist of Point-of-care Ultrasound in Gastrointestinal Stromal Tumors

**DOI:** 10.5811/cpcem.2017.12.36375

**Published:** 2018-01-24

**Authors:** Kim P. Chan

**Affiliations:** Sengkang Health, Department of Emergency Medicine, Singapore

## Abstract

Gastrointestinal stromal tumors (GISTs) are rare, and patients usually present with vague and non-specific abdominal symptoms. This report illustrates how point-of-care ultrasound performed in the emergency setting in the evaluation of such patients helped in management of two undiagnosed GIST patients.

## INTRODUCTION

Gastrointestinal stromal tumors (GISTs) are rare gastrointestinal tumors though they are the most common mesenchymal neoplasms of the gastrointestinal tract. Patients may present with non-specific symptoms (abdominal fullness, pain or discomfort, malaise, or palpable abdominal mass) or may be asymptomatic. Computed tomography of the abdomen and pelvis (CTAP) are crucial in the diagnosis and staging of GISTs but are not universally available in all emergency settings worldwide. Point-of-care ultrasonography (POCUS) has the potential of being the first-line modality in the evaluation of acute patients with such non-specific symptoms. To date, there is no published article on the use of POCUS in the identification or management of GISTs. And presented here are two cases of GIST with different clinical presentations where POCUS had an impact on the management.

## CASE REPORT (ONE)

**Image 1:**
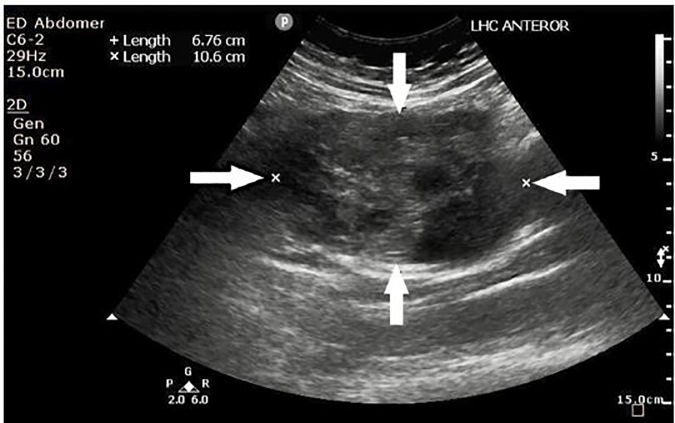
Point-of-care ultrasound of the left hypochondrium showing a heterogeneous mass as outlined by the white arrows, later determined to be a gastrointestinal stromal tumor.

A 64-year-old woman presented to the emergency department (ED) after three episodes of syncope. She also had an increasing abdominal girth with epigastric fullness and discomfort for the prior few months. Clinically her vital signs were blood pressure (BP) 146/97 millimeters of mercury (mmHg), pulse rate of 121 beats per minute (bpm), respiratory rate 18 breaths per minute (BPM), pulse oximetry of 96% on room air and temperature at 36.6 degrees Celsius (C). Her abdomen, though distended, was soft and non-tender. A point-of-care ultrasound (POCUS) was performed to evaluate for possible aortic pathology but revealed a large left hypochrondrial mass of mixed echogenicity (Image 1); intra-abdominal free fluid was noted over the splenorenal and supra-pubic recesses.

**Image 2:**
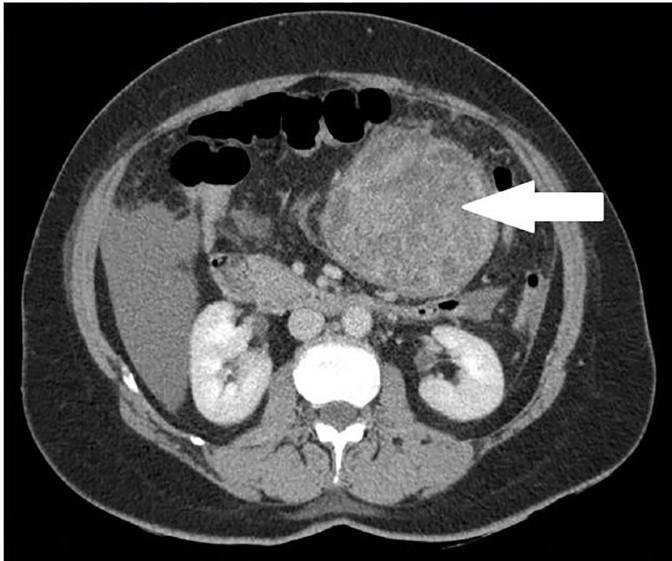
Computed tomography of the abdomen and pelvis at level of renal hilum showing a large, heterogeneous exophytic mass of 9.2 × 9.5 × 9 cm as indicated by the white arrow, later determined to be a gastrointestinal stromal tumor.

**Image 3:**
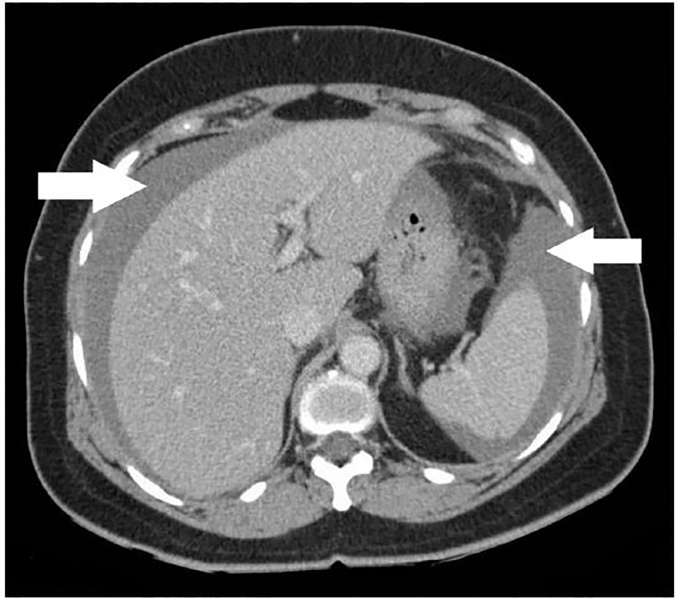
Computed tomography of the abdomen and pelvis showing free intra-abdominal fluid as indicated by the white arrows, later determined to be a gastrointestinal stromal tumor.

CTAP was performed, which showed a large heterogeneous exophytic mass (9.2.× 9.5 × 9 cm) arising from the greater curve of the stomach suspicious of GIST (Image 2), with hemorrhagic free fluid in the abdomen and pelvis (Image 3).

Intra-operative finding revealed a thin-walled stomach GIST with recent bleeding and intraperitoneal hematoma adjacent to the GIST. Histopathological report was that of GIST of intermediate to moderate risk of behavior.

## CASE REPORT (TWO)

**Image 4:**
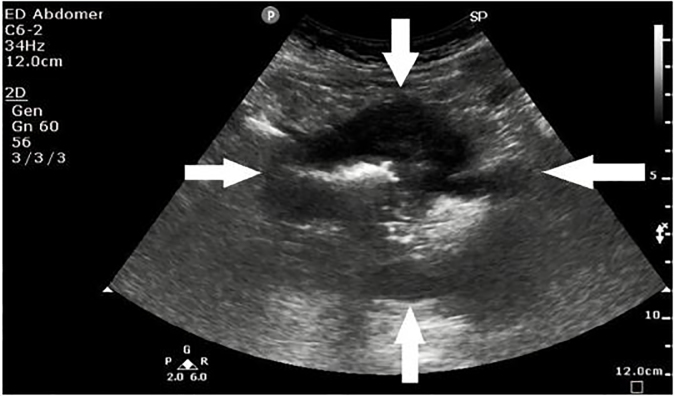
Point-of-care ultrasound of the left lower quadrant showing a suprapubic mass of mixed echogenicity with marginal halo as outlined by the white arrows, later determined to be a gastrointestinal stromal tumor.

A 53-year-old man presented to the ED with a month of lower abdominal discomfort, which was more pronounced when the bladder was full. He had already undergone a cystoscopy and colonoscopy, which did not reveal any significant finding to account for his symptoms. At the ED, his vital signs were BP 122/71 mmHg, pulse rate of 92 bpm, respiratory rate 18 bpm, pulse oximetry of 99 % on room air and temperature at 36.2ºC. Clinical examination of the abdomen revealed a left lower quadrant mass that was mildly tender on palpation. POCUS showed a suprapubic mass of mixed echogenicity with a marginal halo (Image 4).

CPC-EM Capsule

What do we already know about this clinical entity?

Gastrointestinal stromal tumors (GISTs) are rare malignancies of the gastrointestinal tract, which are often incidental findings on computed tomography (CT). Symptomatic patients often present late and the diagnosis would usually involve CT and endoscopic ultrasonography.

What makes this presentation of disease reportable?

Point-of-care ultrasound (POCUS) has never been previously described in the literature as a modality for the diagnosis of GIST. This case report illustrates the usefulness of POCUS in the evaluation of patients with non-specific abdominal symptoms and/or findings, leading to the diagnosis of GIST.

What is the major learning point?

POCUS may be a potential screening modality for patients with non-specific abdominal symptoms and/or findings, leading to the diagnosis of GIST.

How might this improve emergency medicine practice?

POCUS can help emergency physicians to expedite investigations and management plans for patients with non-specific abdominal symptoms and/or findings. This leads to an earlier diagnosis of GIST and the potential to improve the patient’s prognosis.

**Image 5:**
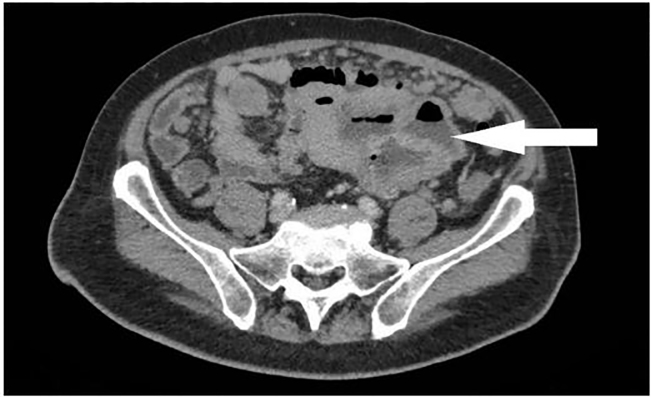
Computed tomography of the abdomen and pelvis showing a centrally necrotic mass of 9.3.x 11.6 × 9.3 cm that was closely related to the small bowel loops as indicated by the white arrow, later determined to be a gastrointestinal stromal tumor.

He underwent a CTAP, which revealed a large centrally necrotic mass (9.3.x 11.6 × 9.3 cm) that was closely related to the pelvic small bowel loops (Image 5), highly suggestive of malignant GIST with peritoneal and liver metastases. A small amount of ascites was noted in the pelvis.

## DISCUSSION

GISTs accounts for 90% of mesenchymal tumors in the gastrointestinal tract and 2–3% of all gastric malignancies.^1^ It can arise anywhere in the gastrointestinal tract with the stomach being the most common site (up to 70%), as well as in extra-visceral locations such as mesentery, omentum or retroperitoneum.^2^ The global incidence of GISTs is reported to be between 10 to 15 cases per million population, with an age range between 10 to 100 years; the median age is reported in the mid-60s and there is equal male-to-female distribution.^3^ Clinical manifestations of GISTs are erratic and depend on their size and location.^1^, ^4^ The diagnosis of GIST is often delayed as small GISTs are often asymptomatic and patients generally present with non-specific symptoms such as early satiety, bloating, abdominal pain and fatigue from anemia.^4^, ^5^ And by the time of presentation, the tumor is often large and has spread to other organs.^6^

The most common symptom at presentation is bleeding,^7^, ^8^ as seen in our first patient who presented with syncope episodes secondary to intra-abdominal bleeding. These two cases illustrate the usefulness of point-of-care ultrasonography in evaluating patients with vague abdominal complaints or a clinically palpable mass in the acute setting. Although endoscopic ultrasonography has been described as a valuable tool that allows diagnosis and localization of GISTs and their characterization such as extra-mural involvement,with the possibility of performing real-time needle biopsy for confirmation,^8^, ^9^ such modality is often not readily available in the ED.

Trans-abdominal sonography can help to characterize the internal content of both the primary and metastatic GIST. It usually appears as a homogenously hypoechoic mass in close relation to the gastrointestinal tract, but a variable degree of heterogeneity is seen in larger GISTs representing necrosis, cystic changes and hemorrhages. The site of origin is often hard to detect on US especially for a large tumor.^8^ Computed tomography (CT) is recommended as the primary imaging modality for the diagnosis of GISTs.^9^ CT can delineate the full extent of a large GIST and detect local invasion and distal metastases.^10^ It provides the basis for diagnosis and staging, and when combined with positron emission tomography, it is the gold standard method for assessment of response to treatment.^11^ However, such modalities are sophisticated and require time and expertise for the imaging and interpretation. In the acute emergency setting, POCUS has an important role as a first-line screening tool in such situations.

## CONCLUSION

Although we are not able to confidently diagnose a GIST based on a point-of-care ultrasound, certain sonographic features, e.g., heterogenicity and marginal halo of an abdominal mass that is not within an intra-abdominal organ such as liver, spleen or kidneys, are suggestive of GIST and would prompt us to arrange for urgent definitive imaging modalities. Our two cases indicate that point-of-care ultrasound may be useful in screening for patients with vague abdominal pain.
